# Discontinuing hormonal gender reassignment: a nationwide register study

**DOI:** 10.1186/s12888-024-06005-6

**Published:** 2024-08-19

**Authors:** Riittakerttu Kaltiala, Mika Helminen, Timo Holttinen, Katinka Tuisku

**Affiliations:** 1 Tampere University, Faculty of Medicine and Health Techonolgy, Tampere University Hospital and Vanha Vaasa Hospital, Tampere, 33014 Finland; 2 Tays Research Services, Wellbeing Services County of Pirkanmaa, Tampere University, Faculty of Social Sciences, Tampere, Finland; 3 Department of Adolescent Psychiatry, Tampere University Hospital, Tampere, Finland; 4 Department of Psychiatry, Helsinki University Hospital, Helsinki, Finland

**Keywords:** Gender dysphoria, Gender reassignment, Masculinizing hormones, Feminizing hormones, Detransition, Register study, Time trend

## Abstract

**Background:**

With increasing numbers of people seeking medical gender reassignment, the scientific community has become increasingly aware of the issue of detransitioning from social, hormonal or even surgical gender reassignment (GR). This study aimed to assess the proportion of patients who discontinued their established hormonal gender transition and the risk factors for discontinuation.

**Methods:**

A nationwide register-based follow-up was conducted. Data were analysed via cross-tabulations with chi-square statistics and t tests/ANOVAs. Multivariate analyses were performed via Cox regression, which accounts for differences in follow-up times.

**Results:**

Of the 1,359 subjects who had undergone hormonal GR in Finland from 1996 to 2019, 7.9% discontinued their established hormonal treatment during an average follow-up of 8.5 years. The risk for discontinuing hormonal GR was greater among later cohorts. The hazard ratio was 2.7 (95% confidence interval 1.1–6.1) among those who had accessed gender identity services from 2013 to 2019 compared with those who had come to contact from 1996 to 2005. Discontinuing also appeared to be emerging earlier among those who had entered the process in later years.

**Conclusions:**

The risk of discontinuing established medical GR has increased alongside the increase in the number of patients seeking and proceeding to medical GR. The threshold to initiate medical GR may have lowered, resulting in a greater risk of unbalanced treatment decisions.

**Trial registration number (TRN):**

Not applicable (the paper does not present a clinical trial).

## Background

In gender medicine, transition refers to people with sex-discordant gender identities making changes in their lives to live in their experienced gender, socially (appearance, name, personal pronouns), juridically (identity documents) or medically (hormonal and surgical medical interventions that modify secondary sex characteristics*)*. Detransition refers to people aborting their initiated transition and reversing it, totally or partially, to live in a sex-accordant role by reversing the abovementioned steps of transition.

Recent decades have witnessed an exponential increase in those seeking medical interventions to support their transition (medical gender reassignment, GR), with an increasing share of younger individuals of the female sex [1, 2]. Psychiatric morbidity among people who contact specialized gender identity services (GISs) has increased simultaneously [2, 3] and is particularly pronounced among the youngest age groups [4].

It has long been assumed that very few patients embarking on medical GR regret their choice and seek to reverse it. From the 1970s to the 2010s, estimates of those regretting their initiated GR were only in the region of 2% [5, 6]. However, more recent research suggests that alongside the increase in the number of people accessing medical gender reassignment, reversing the initiated transition seems to be increasing [7]. In recent samples, 20–30% of those who initiated hormonal GR discontinued hormonal treatment in four to five years [8, 9]. It is possible that some patients discontinue hormonal treatment because they have reached their transition goals. Some changes, such as lowering of the voice, can be reached with relatively short hormonal treatments and are permanent, while maintaining some other changes require permanent treatment.

People abandoning their gender transition have reported various reasons for doing so, such as coming to terms with their natal sex, concerns about medical complications, attributing gender dysphoria to reasons other than gender identity, such as trauma or mental disorders, finding that the transition did not alleviate distress, struggles with sexual orientation and discrimination [10, 11]. More importantly, those who have detransitioned have repeatedly reported that before their embarking on medical GR, insufficient attention was given to their mental health and psychosocial problems, which, in retrospect, they believed played a major role in their desire to transition. They have expressed concerns that assessments for medical gender reassignment were too superficial, with no search for explanations for their distress beyond an assumed stable sex-discordant identity requiring transition. [10, 11]. This contradicts calls to lower the threshold for medical gender reassignment [12, 13]. Several recent national guidelines and recommendations [4, 14, 15], however, emphasize the appropriate treatment of psychiatric comorbidities and associated difficulties as well as a psychosocial intervention facilitating identity exploration as first-line interventions for gender dysphoria before considering medical interventions, particularly for young people.

In Finland, gender identity assessments potentially leading to medical GR interventions are conducted at two of the country’s five university hospitals. Services for legal adults (> 18 years) have been available since the early 1990s [16] and became available to minors in 2011 [17]. The national guidelines require minors presenting with feelings of gender dysphoria to first undergo psychosocial intervention to support identity exploration and to receive appropriate treatment for any severe mental disorders [14], after which they can proceed to the centralized GIS, where diagnostic assessments are carried out by specialized mental health teams. Both GISs have separate diagnostic teams for minors and for adults. Hormonal GR interventions are initiated at the same hospitals in gynecological outpatient clinics, and after stabilization, hormonal treatment is transferred to services in the patients’ places of residence. Genital surgeries with gender identity indication are nationally centralized to one university hospital and require recommendations from both nationally centralized diagnostic GIS units. Psychiatric treatment for any concomitant mental health condition is provided at the specialized secondary care or primary health care facility in the patient’s place of residence. Until 2022, diagnostic assessments at the nationally centralized GIS were also a prerequisite for registered sex change, but since 3 April 2023, legal adults have been granted legal GR on the basis solely of their own request. Medical GR remains nationally centralized and is available case-by-case after a comprehensive diagnostic assessment by a multidisciplinary mental health team, as outlined in the national guidelines [14, 18, 19].

An important ethical principle in all medicine is to not harm. A more severe or life-threatening condition may justify greater risks in its treatment. In medical gender reassignment, hormonal and surgical interventions are performed on physically healthy bodies. If the patient subsequently regrets the changes brought by the treatments, not to mention undesired side effects, this can be considered harmful. As in other Western countries, alongside the vastly increasing number of referrals to the GIS, increasing numbers of younger people with increasingly common psychiatric needs have initiated medical GR in Finland [2]. This may be followed by increasing numbers of people who later feel otherwise about their medical GR. On the other hand, the purpose of the nationally centralized and comprehensive assessment before medical GR is to ensure reasoned treatment decisions and satisfactory patient outcomes, avoiding possible regrets. This may counteract the risks related to the more complex presentations among those seeking medical GR. Those abandoning their gender transitions have repeatedly claimed that the distress accompanying their situation is not appropriately addressed [20]. It is crucial to take seriously the desire to reverse medical GR and to ascertain its likelihood and predictors to target medical GR safely and provide appropriate services for those opting out of treatment that has resulted in irreversible changes in a healthy pretreatment body. In the present study, we referred to national registry data to determine which patients are likely to discontinue hormonal GR. More specifically, we asked:

1. How commonly did people who proceeded to hormonal GR after assessment in the nationally centralized GIS from 1996 to 2019 discontinue their established hormonal GR?
2. What are the predictors of discontinuation in terms of age, age at admission to the GIS, direction of transition, surgical treatment, psychiatric treatment needs and cohort effects?
3. Has the risk of discontinuing hormonal GR changed over time?

## Methods

### Design and setting

A register-based follow-up study was conducted using information held in health care registers in Finland. These comprehensive and reliable national registers can be used to study large patient groups and collate information from different registers (on an individual level) via the unique personal identity code assigned to each permanent resident of Finland. Register data can be applied for research purposes from the Finnish Social and Health Data Permit Authority Findata and Statistics Finland. Data extraction, linkages and pseudonymization are carried out by these authorities, and researchers are allotted a special secure connection for pseudonymized data only. Analyses producing unduly precise information potentially enabling a person to be identified must be amended to ensure the anonymity of the persons included. The present study obtained ethical approval from the ethics committee of Tampere University Hospital (R20040R) and relevant permissions from Findata (THL/5188/14.02.00/2020) and Statistics Finland (TK/1016/07.03.00/2020). In accordance with Articles 6e and 9i and j of Regulation (EU) 2016/679 of the European Parliament and of the Council [21], no individual informed consent was needed.

A personal identity code is assigned at birth (or upon obtaining Finnish citizenship). This indicates sex (male or female). Legal sex change entails a new identity code. People are listed in the national registers according to their currently valid personal identity code. This code serves to retrieve data from various registers (including earlier data under the original identity code). Researchers cannot obtain information about identity code changes (changes in juridical sex). Researchers using the data never see the actual identity codes.

### Data extraction

Subjects referred to either of the two nationally centralized GISs were identified from the hospital databases of Tampere and Helsinki University Hospitals. The first contact with a diagnostic team in either of the two GISs was recorded as the index date. The Finnish Social and Health Data Permit Authority Findata combined the lists from the two hospitals. A total of 3,665 individuals were identified as having contacted the nationally centralized gender identity units between 1996 and 2019. Of these, 1,359 had initialized and embarked on feminizing or masculinizing hormonal treatment (see below, next paragraph) and formed the subjects of the present study.

The register of the Social Insurance Institution of Finland (KELA), with information on prescription medications purchased and information on reimbursement, was used to obtain information on hormonal GR in the clinical GD group. Persons diagnosed with F64.0 (since 2020, also F64.8) in the nationally centralized gender identity units are entitled to special reimbursement (code 121) for their hormonal treatment, as are patients suffering from specified endocrine disorders. In the treatment of gender dysphoria, special reimbursement is available when hormonal treatment has continued for more than a year. The data on prescription medications were collected up to the end of 2021.

The Care Register for Health Care [22] was used for information on all treatment contacts to specialist-level psychiatric services from 1994 to 2022. The register, which has been in operation since 1994, includes all outpatient and inpatient contacts with specialist-level health services in Finland. For all contacts, admission and discharge dates were extracted. The Care Register for Health Care was further used to provide information on gender reassignment surgeries.

The Population Register provided information on those deceased and their dates of death.

Measures.

### Discontinuing hormonal GR

Subjects entitled to special reimbursement for hormonal treatments were considered to have discontinued their hormonal GR if they had purchased no hormones for more than 12 months before the end of the data collection or, if deceased, for 12 months or more before their death, or if they had been purchasing specially reimbursed feminizing hormones but had later switched to masculinizing hormones, or vice versa. To obtain reimbursements for prescription medications from the Social Insurance Institution of Finland (KELA), these medications can be purchased for only three months at a time. Thus, not purchasing them for over a year means that they are most likely not being taken. The last date of purchase of the originally prescribed hormonal GR medication was recorded. Patients who discontinue hormonal GR may require birth-sex accordant hormonal replacement to detransition after gonad removal or if their natural hormone production does not resume. For subjects whose specially reimbursed hormone treatment had changed from masculinizing to feminizing or vice versa, the last date of purchase of the originally initiated type of hormonal GR was recorded.

### Types and durations of hormonal GR

In the analyses, hormonal GR was divided into feminizing and masculinizing. The duration of hormonal GR with special reimbursement was calculated in months from the dates of first and last/latest purchase of the originally initiated masculinizing/feminizing hormones.

### Time variables

The subject’s year of birth was used in the analyses as a continuous variable. The year of initial contact with the GIS (index year) was categorized into intake cohorts with the first contact with the GIS in 1996–2005 vs. 2006–2012 vs. 2013‒2019. As the inclusion period did not fall into three even periods, the first period, with a clearly lower case load, was extended.

### Age

Age at first contact with the GIS (index date) was calculated from the dates of index contact and birth. Age in years was used in bivariate analyses as a continuous variable. In multivariable analyses, age was divided into adolescent (up to 22 years old) and adult (23+) at index contact.

### Gender reassignment surgeries

The gender reassignment surgeries recorded were genital surgery (vaginoplasty, phalloplasty/metoidioplasty) and chest masculinization.

### Specialist-level psychiatric treatment contact

Specialist-level psychiatric treatment contacts other than those related to gender identity assessment were recorded. Having received specialist-level psychiatric treatment was used in the analyses as a comprehensive dichotomous variable (yes/no). Furthermore, having specialist-level psychiatric treatment contact before entering the GIS (yes/no) was used, as was having specialist-level psychiatric treatment two or more years after entering the GIS (yes/no).

Statistical analyses.

Bivariate associations between discontinuing hormonal GR and the explanatory variables were studied via cross-tabulations with chi-square statistics (Fisher’s exact test where appropriate) and the Mantel‒Haenszel test for categorical variables and t tests and ANOVA for continuous variables. Multivariate associations were studied via Cox regression, accounting for differences in follow-up times. Discontinuing hormonal GR was entered as the dependent variable. The independent variables entered were (1) direction of hormonal treatment (masculinizing/feminizing), year of birth and index year cohort; (2) GR surgeries; (3) age at first entering the GIS (adolescent vs. adult); and (4) and, finally, having received specialist-level psychiatric treatment (yes/no). Hazard ratios (HRs) with 95% confidence intervals are given. The cut-off for statistical significance was considered *p* < 0.05.

## Results

### The sample

There were 1,359 people who, after having been assessed in the nationally centralized GIS, had purchased masculinizing or feminizing hormones with a special reimbursement code. The mean (sd) age of the participants on admission to the GIS was 25.6 (9.3) years, and 49.1% of them were under 23 years of age. In total, 467 (34.4%) had received feminizing treatment, and 892 (65.6%) had received masculinizing treatment. At index contact with the GIS, those who subsequently initiated feminizing GR were older than those who proceeded to masculinizing GR (29.7 (11.1) vs. 23.4 (7.3) years, *p* < 0.001). The mean (sd) duration of hormonal GR was 62.0 (57.0) months, with a median of 44.5 months, with no difference between masculinizing and feminizing treatments. Genital surgeries were more commonly performed on those who had proceeded to feminizing treatment (46.7% vs. 14.9%, *p* < 0.001). Among those on masculinizing treatment, 41.5% had undergone chest masculinization. Among all patients proceeding to hormonal GR, 57.4% had ever had treatment contact with specialist-level psychiatric care.

### Discontinuing hormonal GR

A total of 107 subjects (7.9% of those who had started hormonal GR and obtained special reimbursement for it) had not been purchasing GR hormones for at least a year before the end of data collection (or before the subject died) or had changed from feminizing GR to masculinizing treatment, or vice versa. These were considered to have discontinued hormonal GR. Among those who had obtained feminizing GR, 10.5% had discontinued hormonal treatment, and among those who had obtained masculinizing GR, 6.5% (*p* = 0.004). Those who discontinued hormonal GR were slightly older at the index contact and at their latest purchase of specially reimbursed hormones than those who continued hormonal GR. The two groups had used hormonal GR for comparable periods. Those who discontinued and those who stayed on hormonal GR had comparable specialist-level psychiatric treatment contacts. (Table 1)

Those who discontinued and those who continued hormonal GR had equally common specialist-level psychiatric treatment contact before contacting the GIS (15.3% vs. 17.8%, *p* = 0.5) as well as two or more years after entering the GIS (59.9% vs. 57.0%, *p* = 0.2).

**Table 1:** Follow up times, type (feminizing/ masculinizing) of prescribed and continued/ discontinued hormonal gender reassignment, timing and duration of contacting gender identity services and using hormonal GR, and specialist level psychiatric treatment contact among subjects who continued/ discontinued their established hormonal gender reassignment. Distributions (%, n/N) of the categorical variables and mean(sd) of the continuous variables, and their comparison between the intake cohorts

	All, index date	Index date	Index date	Index date	p (between cohorts)
	1996–2019	1996–2005	2006–2012	2013–2019	
Follow-up-time from index contact to 31.12.21, mean(sd) in years					
	8.5 (4.3)	20.3 (4.0)	11.7 (1.6)	6.0 (1.6)	
Specially reimbursed hormonal GR (n)	1359	102	438	819	
Specially reimbursed hormonal GR was (% (n/N)					0.002*
towards female	34.4% (467/1359)	41.2% (42/102)	39.0% (171/438)	31.0% (254/819)	
towards male	65.6% (892 /1359)	58.8% (60/102)	61.0% (267/438)	69.0% (565/819)	
Had discontinued using in 31.12.21 (n)	107	24	45	38	
Discontinued hormonal GR (% (n/N)) was					0.003*
towards female	45.8% (49/107)	70.8% (17/24)	44.4% (20/45)	31.6% (12/38)	
towards male	54.2% (58/107)	29.2% (7/24)	55.6% (25/45)	68.4% (26/38)	
Age at index contact, years (mean(sd))					
continued hormonal GR	25.3 (9.0)	30.2 (7.8)	25.6 (9.1)	24.6 (9.0)	< 0.001
discontinued hormonal GR	29.4 (10.7)	34.0 (7.9)	27.2 (10.2)	29.0 (11.9)	0.04
p	p < 0.001	p = 0.04	p = 0.3	p = 0.03	
Age at latest purchase of hormones, years (mean(sd)					
continued hormonal GR	34.3 (10.5)	50.7 (8.6)	37.7 (9.6)	30.9 (9.0)	< 0.001
discontinued hormonal GR	37.7 (11.6)	47.9 (7.5)	35.7 (10.4)	33.7 (11.5)	< 0.001
p	p = 0.001	p = 0.2	p = 0.2	p = 0.07	
Months on hormonal GR (mean(sd); median)					
continued hormonal GR	61.6 (57.0); 44	209.7 (59.3); 207	94.8 (35.2); 95	30.1 (22.1); 27	< 0.001
discontinued hormonal GR	66.6 (58.0); 48	120.6 (58.6); 128	65.9 (41.8); 57	33.3 (43.6); 16	< 0.001
*p*	p = 1.0	*p < 0.001*	*p < 0.001*	*p = 0.6*	
Specialist level psychiatric treatment contact					
continued hormonal GR	56.5% (708/1252)	57.7% (45/78)	63.4% (249/393)	53.0% (414/781)	0.003
discontinued hormonal GR	67.3% (72/107)	66.7% (16/22)	66.7% (30/45)	68.4% (26/38)	1
p	p = 0.03	p = 0.5	p = 0.7	p = 0.07	

*Mantel-Henzel test for linear association

### Changes across intake cohorts

The basic characteristics of the subjects changed across intake cohorts. The mean (sd) age among those who had contacted the GIS from 1996 to 2005 and subsequently proceeded to hormonal GR was 31.1 (7.9); from 2006 to 2012, it was 25.7 (9.3); from 2013 to 2019, it was 24.8 (9.2) years (*p* < 0.001); and the proportion of adolescents (< 23-year-olds) was 13.7% vs. 48.9% vs. 53.6% (*p* < 0.001). The proportion of those seeking change towards masculinity increased, and the same change was observed among those discontinuing hormonal GR. The proportion of those with specialist-level psychiatric treatment contacts fluctuated between cohorts among those continuing hormonal GR but remained unchanged among those who discontinued it (Table 1).

### Multivariable analyses

The hazard ratio (HR) for discontinuing hormonal GR was greater among those in the latest intake cohort (2013–2019) as compared to those in the earliest cohort (1996–2005) when the type of hormonal GR (masculinizing vs. feminizing) and year of birth were accounted for (Table 2 Model 1) and when surgical GR (Table 2 Model 2), age at index admission (adolescent vs. adult) (Table 2 Model 3) and, finally, specialist-level psychiatric treatment contact (Table 2 Model 4) were added. Genital surgeries were associated with a decreased HR for the discontinuation of hormonal GR. Earlier year of birth was very slightly but statistically significantly associated with increased HR for discontinuing hormonal GR in the first models but levelled out in subsequent models.

**Table 2:** Predictors of desisting from hormonal gender reassignment in multivariable models (Cox regression). Hazard ratios (HR) with 95% confidence intervals (CI) are reported

	Model 1: type of hormonal GR, birth year, index year		Model 2: add surgical GR		Model 3: add age group at entering gender identity assessment		Model 3: add psychiatric treatment	
Hormonal GR								
masculinizing	ref		ref		ref		ref	
feminizing	1.3 (0.9-2.0)	0.2	1.4 (0.9–2.1)	0.1	1.4 (0.9–2.2)	0.2	1.3 (0.9–2.4)	0.2
birth year	**0.98 (0.96-1.0)**	**0.04**	**0.98 (0.96-1.0)**	**0.03**	1.0 (1.0–1.0)	0.2	1.0 (1.0–1.0)	0.1
index year at								
1996–2005	ref		ref		ref		ref	
2006–2012	1.6 (0.8–3.1)	0.1	1.4 (0.7–2.7)	0.2	1.3 (0.7–2.7)	0.4	1.4 (0.7–2.7)	0.4
2013–2019	**3.1 (1.4–6.9)**	**0.007**	**2.5 (5.1–5.8)**	**0.03**	**2.5 (1.1–5.7)**	**0.03**	**2.7 (1.1–6.1)**	**0.02**
genital surgery	-	-	**0.4 (0.3–0.7)**	**< 0.001**	**0.4 (0.3–0.7)**	**< 0.001**	**0.5 (0.3–0.7)**	**0.01**
chest masculinization	-	-	0.8 (0.5–1.4)	0.4	0.8 (0.5–1.4)	0.4	0.8 (0.4–1.3)	0.3
age group at index	-	-	-	-	1.2 (0.7-2.0)	0.6		
adolescent (until 22 yrs)							ref	
adult (23 + yrs)							1.2 (0.7-2.0)	0.6
specialist level psychiatric treatment contact*	-	-	-	-	-	-	1.4 (0.9–2.2)	0.1

*Findings did not change when specialist level psychiatric treatment contact beyond 2 years after entering GIS was used instead of contact at any time

### Confirmatory analyses

Because the oldest individuals in the sample may have discontinued hormonal GR due to reaching the age of natural decline in hormonal levels, the final model was repeated among individuals younger than 60 at the end of data collection, but this did not change the findings.

A further confirmatory analysis was carried out using data from those subjects whose index contact was before 2018 because of the rather short follow-up times among those who had started their gender identity assessments in 2018 or 2019. This caused no changes to the findings presented in Table 2.

### Changes in the discontinuation of hormonal GR over time

**Fig. 1:**
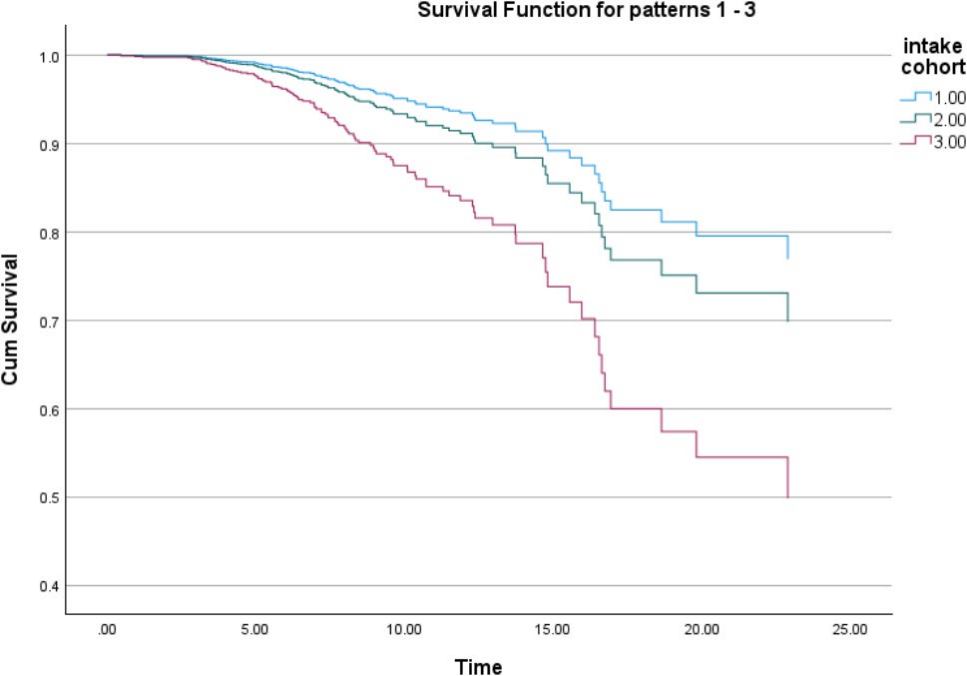
Time (in years)* to discontinuing hormonal gender reassignment in the different intake cohorts (1 = 1996–2005, 2 = 2006–2012, 3 = 2013–2019). *modeled by Cox regression

Survival curves for the three index date cohorts suggested that the discontinuation of hormonal GR emerged in a shorter time from the earliest to the latest intake cohort (Fig. 1). To explore this further, discontinuation within two years of obtaining special reimbursement for hormonal GR was scrutinized among those with index dates before 2018. Among the two earlier intake cohorts (combined due to small cell frequencies in the original categories), 1.3% of those who had started hormonal GR discontinued it within two years; among the latest intake cohort, 2.9% (*p* = 0.06).

## Discussion

In this nationally representative register study covering subjects proceeding to hormonal GR over three decades, 7.9% discontinued their established hormonal GR. The risk for discontinuing hormonal GR was greater in the latest intake cohort (2013–2019) than in the earliest cohort (1996–2005). Genital surgeries were associated with a decreased risk of discontinuing hormonal GR. Over the decades, the time to discontinuation grew shorter.

The proportion of those who discontinued treatment was smaller than that reported in the most comparable study [9] from the USA, where almost one-third of adolescents and young adults discontinued their hormonal GR within four years. The relatively low discontinuation rate in our study may be due to the comprehensive assessment in the nationally centralized GIS before initiating hormonal treatments. When severe psychiatric comorbidities are present, great care is taken in considering physical interventions [2, 14, 17]. The proportion of those who discontinued their established hormonal GR was nevertheless manifold compared with earlier reports of proportions regretting medical transition among samples who had initiated their treatments between the 1960s and 2010s [5, 6]. However, both of those reports focused on actively expressed regrets, and in the latter study [6], the proportion lost to follow-up—with later development thus unknown—was high. The proportion discontinuing their established hormonal GR in the present study was comparable to the proportion defined as detransitioners (those who discontinued treatment and reverted to living in their original gender role) in a register-based study of 175 subjects initially assessed in 2017–18 in the UK [7]. However, in that UK study, a clearly greater additional share of the studied group also subsequently disengaged from the treatments or did not adhere to their treatment plan. In a study evaluating the situation of people diagnosed with GD in a specified GP practice population [8] and, as noted, in a register study in the USA [9], much greater shares discontinued their medical GR. Direct comparisons among these studies are not feasible because of their different focuses and methodologies. However, together with the most recent studies, our study suggests that discontinuing hormonal GR is a significant phenomenon in gender medicine, and studies reporting the experiences of detransitioners [10, 11] suggest that it is often related to profound psychological distress.

In multivariate models accounting for differences in follow-up times and for changes in patient characteristics across intake cohorts, the risk of discontinuing hormonal GR was almost threefold among those patients who had contacted the GIS from 2013 to 2019 compared with those who had contacted the GIS from 1996 to 2005. Our findings also suggest that the time to discontinuation of hormonal GR may have shortened among the later patients; however, in the latest intake cohort, more discontinuations may still emerge, and this will eventually affect the final conclusions about the average time to discontinuation. The proportion of subjects who discontinued after short use, a maximum of two years of specially reimbursed medication use, nevertheless appeared to have increased. (This will mean a maximum of three years of total use, given the rules on special reimbursement). Over the whole study period, the number of people seeking GR increased manifoldly [2], as did the number of subjects proceeding to hormonal GR. Alongside with this, the risk of discontinuing established medical GR has also increased. The populations seeking medical GR may have changed in a way that limits positive treatment outcomes. It is already known that subjects currently seeking medical GR are, unlike earlier, predominantly birth-registered females, who are younger than before and present with more psychiatric comorbidities than before [1–3, 20]. These observations may suggest that an increasing share of GD patients actually do not present with achieved, consolidated identity [20, 23]. In particular, medical transition early in terms of identity development may increase the risk of unbalanced treatment decisions, and this risk appears to have increased towards the present day, with detransitioning as the next step. Greater attention to gender identity issues and GR in the media and social media as well as assertive advocacy for medical GR may play a role in these developments [20, 24, 25].

Somewhat unexpectedly, the need for specialist-level psychiatric care did not differentiate those who continued and those who discontinued hormonal GR. Approximately one in six of the patients who had started hormonal GR, both those who later discontinued and those who continued the treatment, had needed specialist-level psychiatric treatment before embarking on gender identity assessments. This number was clearly less than that of all patients who were in contact with the GIS [2]. It is expected that the two groups would be comparable at the time of the decision to initiate medical GR and suffer fewer psychiatric comorbidities than those who could not start medical GR. However, psychiatric treatment needs increased vastly after the index contact with the GIS in both groups who proceeded to medical GR, those who subsequently discontinued it and those who continued on hormonal GR. A more detailed analysis of the nature of psychiatric needs and subsequent identity struggles is needed to better understand the discontinuation of medical GR in the future. According to the multivariable analyses, the risk for discontinuing hormonal GR did not differ between those who had initially contacted the GIS during adolescence (< 23 years) and those who had contacted in adulthood. This may be due to assessments being particularly cautious with younger patients, whereas with middle-aged subjects, self-determination may be accorded greater significance.

Having undergone genital surgeries was predictive of a decreased risk of discontinuing hormonal treatments. This may be due to strict treatment protocols requiring psychological stability as part of eligibility for genital surgeries. A recommendation letter is required from both the nationally centralized GIS for gender surgeries to ensure both the patient’s capacity to consent and that their psychological and psychosocial resources will suffice to recover from major surgery.

Methodological considerations.

A strength of the present study is the use of nationwide registry data over three decades. The registers are comprehensive since treatment providers are required by law to report to them all the information on which this study relies. The subjects were identified in the databases of the hospitals where the nationally centralized GISs operate, thereby ensuring the reliability of sampling. The long inclusion period made it possible to analyse changes over time. A limitation is that only subjects who had obtained the special reimbursement code for their hormonal GR were included. There may be subjects who discontinued hormonal GR before their entitlement to special reimbursement (which can take place after a year), and their number is not known. Another limitation is that registers include no information on the reasons for discontinuing hormonal GR. Given the ample publicly funded health services and the special reimbursement for hormonal GR, financial problems are an unlikely reason. Further changes in identity, medical complications or concerns over them, not being helped by GR or social reasons, may contribute [10, 11, 20]. It is also possible that some achieved their goals and therefore discontinued, although this seems implausible in the case of discontinuation after many years. A more profound understanding of the reasons for discontinuing medical GR will require studies using information elicited directly from patients. A further limitation is that regarding the need for psychiatric treatment, this research focused on specialist-level service contacts reflecting severe psychiatric needs. Mild to moderate mental disorders are treated in primary health care. Thus, the need for psychiatric treatment was likely somewhat underestimated in the present study. A limitation is that the possible use of hormonal GR through unofficial routes was not addressed. Publicly funded medical GR interventions are possible only through nationally centralized gender identity services. Obtaining hormonal GR via unofficial routes would likely be related to medical GR not being considered timely in the official treatment route. This finding may suggest that the discontinuation of hormonal GR can be more common among those who obtain hormones unofficially. We combined minors (< 18 at intake to the GIS) and late adolescents (18–22-year-olds at intake) because before 2011, minors entered the assessments only occasionally. Brain development, personality development and identity consolidation continue well beyond the age of reaching legal adulthood [23, 26–30]. Finally, discontinuing hormonal GR, desisting from identifying in a sex-discordant way, detransitioning and regretting medical GR are concepts referring partly to the same phenomenon but not totally overlapping [20]. A register-based study cannot reach these nuances.

## Conclusion

Discontinuing established medical GR appears to be less common in Finland than reported elsewhere. This is likely due to careful, comprehensive assessment before initiating physical treatments. The risk of discontinuing established medical GR has nevertheless increased alongside increases in the number of patients seeking and proceeding to medical GR. In later intake cohorts, discontinuation also appears to emerge earlier. The threshold to initiate medical GR may have decreased, resulting in greater risks of suboptimal decisions. More research is needed on practically all aspects of detransitioning from medical GR.

## Data Availability

The authors are not allowed to give the data to any party. Information about how to apply Finnish register data for research purposes can be found in www.findata.fi.

## Abbreviations

GD: Gender dysphoria
GIS: Gender identity service
HR: Hazard ratio
CI: Confidence interval
